# Microbiological Safety of Street-Vended Beverages in Chow Kit, Kuala Lumpur

**DOI:** 10.3390/ijerph16224463

**Published:** 2019-11-13

**Authors:** Nur Syakirah Mohd Nawawee, Nur Faizah Abu Bakar, Siti Shahara Zulfakar

**Affiliations:** 1 Environmental Health and Industrial Safety Program, Faculty of Health Sciences, Universiti Kebangsaan Malaysia, Kuala Lumpur 50300, Malaysia; nursyakirah_mn@yahoo.com; 2 Center of Applied Health Sciences, Faculty of Health Sciences, Universiti Kebangsaan Malaysia, Kuala Lumpur 50300, Malaysia; nurfaizah@ukm.edu.my; 3 Environmental Health and Industrial Safety Program / Center of Applied Health Sciences, Faculty of Health Sciences, Universiti Kebangsaan Malaysia, Kuala Lumpur 50300, Malaysia

**Keywords:** street vendor, beverage, food, bacterial contamination

## Abstract

Improper handling, poor hygienic practices, and lack of environmental control affect the safety of street-vended beverages. The objective of this study is to determine the bacterial contamination level of three types of beverages (cordial-based drinks, milk-based drinks, fruit juices) sold by street vendors at Chow Kit, Kuala Lumpur. A total of 31 samples of beverages were analyzed to determine total viable count (TVC), total coliform, *Escherichia coli*, and *Staphylococcus aureus* counts via the standard plate count method. The results showed that only 9.7% of the total samples were not contaminated with the tested microorganisms. All milk-based drink samples were positive for TVC and also had the highest average bacterial counts at 5.30 ± 1.11 log Colony Forming Unit/mL (CFU/mL). About 71% of the samples were contaminated with total coliform with the average readings ranging between 4.30 and 4.75 log CFU/mL, whereas 58.1% of the samples were positive with *S. aureus*, with fruit juices having the highest average reading (3.42 ± 1.15 log CFU/mL). Only one sample (milk-based drink) was *E. coli* positive. This study showed that the microbiological safety level of street-vended beverages in Chow Kit, Kuala Lumpur was average and needs to be improved. Provision of food safety education and adequate sanitary facilities at vending sites are suggested to increase the safety of food products.

## 1. Introduction

Street foods are food or drinks, including ready-to-eat snacks sold by hawkers or street vendors for immediate consumption without any further processing [1]. Food sold by street vendors is highly accepted by consumers especially in developing countries [2]. The food industry’s rapid development has introduced various street-vended food options which subsequently increase the consumers’ preferences to dine outside compared to cooking their meals at home [3]. In Malaysia, street-vended food and beverages are well received by consumers because of their taste, low price, and constant availability at any time. [4]. Chow Kit is located in the middle of Kuala Lumpur city in Malaysia and is known among locals and tourists for its wet markets, night markets, and trading bazaars. Street vendors selling local food and beverages are abundant in this area to cater to consumers who come to Chow Kit to shop.

Consumption of roadside foods and beverages can potentially increase the risk of foodborne diseases caused by a wide variety of pathogens [5,6]. Inadequate facilities, lacking environmental control surrounding the vending sites and the absence of food safety behavior among the street-food handlers contribute to the risks of foodborne diseases to the consumers [3,7].

In addition, the location of the vendors by the sides of overcrowded roads with high traffic that contributes to the dispersion of airborne contaminant particles or near waste dumping sites, seem to add to the contamination [2]. Wastewater and waste dumped everywhere on the street vending site attract the presence of vermin such as rats, cockroaches, and flies. Therefore, foods and beverages are hardly protected from dust and flies that may be an intermediary to harmful pathogens to humans [3]. Due to its scarcity of facilities around the vending site, water used for the production of street-vended beverages can also be a significant source of microbial contamination, such as total coliforms, fecal coliforms, fungal, streptococci, and other contaminations [8,9].

This study was conducted to determine the level of bacterial contamination in beverages sold around the Chow Kit area in Kuala Lumpur, Malaysia. The level of total viable bacteria, total coliform, *Escherichia coli* (*E. coli*), and *Staphylococcus aureus* were enumerated.

## 2. Materials and Methods

### 2.1. Sample Collection

Sampling activities were conducted at Chow Kit, Kuala Lumpur area within 1 km distance from the research laboratory. There were only 17 street vendors that sold the tested beverages within the sampling location. All the beverages were displayed in large plastic containers before they were transferred into small plastic cups or tied plastic bags when purchased by customers. Street vendors that sold canned, bottled, or packaged drinks were not included in this study. The business registration status of the sampled vendors was not known and excluded from the scope of this study. A total of 31 beverage samples were purchased from the vendors in their usual packaging, kept in an icebox and maintained at 4 °C during transportation to the laboratory. Out of 31 samples, 11 samples were cordial-based drinks, 9 samples were milk-based drinks, and the other 11 samples were fruit juices. The samples were analyzed within an hour after collection [6]. All samples were analyzed to determine total viable count, total coliform, *E. coli*, and *S. aureus* using the standard plate count [10].

### 2.2. Microbiological Analysis

The samples were transferred into sterile closed containers and gently homogenized using a vortex (BV1005-E, Benchmark Scientific, NJ, USA). A 10-fold serial dilution was performed on the homogenized samples. Then 0.1 mL of appropriate dilutions were plated in triplicates on Plate Count Agar (PCA) (Merck, Darmstadt, Germany), Brilliance ™ *E. Coli*/Coliform Selective Medium Agar (Brilliance) (OXOID, Hampshire, United Kingdom), and Mannitol salt agar (MSA) (OXOID, Hampshire, United Kingdom) for enumeration of the total plate, total coliform, *E. coli*, and *S. aureus*, respectively. All the plates samples were then incubated aerobically at 37 °C for 24 h. Formed bacterial colonies on tested media were enumerated and presented as log CFU/mL. Presumptive *E. coli* and *S. aureus* were then subjected to a series of standard biochemical tests for further confirmation.

### 2.3. Statistical Analysis

Microbial counts (log CFU/mL) were presented as mean ± standard deviation (SD). One sample t-test was conducted to compare the bacterial level in the tested beverages with the Malaysian standards: specification for ready-to-drink beverages (carbonated and non-carbonated) (MS601:1994) [11], World Health Organization (WHO) Guidelines for Drinking-Water Quality [12], and Food Standards Australia New Zealand (FSANZ) Compendium of Microbiological Criteria for Food [13]. One-way ANOVA was also conducted to compare the level of bacterial contamination between the three types of beverages (milk-based drinks, fruit juices, cordial-based drinks). All statistical tests were performed using SPSS version 23.0 (IBM Corp., Armonk, NY, USA). The results were considered significant at *p* < 0.05 unless otherwise stated.

## 3. Results

The microbial loads of total viable counts (TVC), total coliform, *E. coli*, and *S. aureus* are summarized in Table 1. Based on the results, 90.3% of total samples were contaminated with viable bacteria with an average reading of 5.03 ± 0.23 log CFU/mL. All milk-based drinks were found to be contaminated and had the highest mean total viable count of 5.30 ± 1.11 log CFU/mL whereas 90.9% of fruit juices (4.90 ± 0.87 log CFU/mL) and 91.8% of cordial based-drinks (4.90 ± 1.42 log CFU/mL) were found to be contaminated. About 71% of the total samples showed a positive presence of total coliform with fruit juices showing the highest average reading at 4.75 ± 0.79 log CFU/mL, followed by cordial-based drinks at 4.45 ± 0.75 log CFU/mL. Although milk-based drinks showed the lowest mean total coliform counts (4.30 ± 1.13 log CFU/mL), all of the samples were positive with the presence of this microorganism. Meanwhile, only one sample (milk-based drink) was found to be *E. coli*-positive (3.05 ± 0 log CFU/mL). *S. aureus* was detected in 58.1% of the total samples. Milk-based drinks had the highest *S. aureus*-positive samples (77.8%) with also the highest average reading of 3.29 ± 0.86 log CFU/mL compared to other types of beverages, whereas cordial-based drinks had the lowest positive sample (45.5%) and also the lowest mean bacterial count at 2.82 ± 0.46 log CFU/mL.

Malaysian specifications for ready-to-drink beverages (carbonated and non-carbonated) (MS601:1994) [11] was used as a guideline for the permissible total viable count (3.70 log CFU/mL) and total coliform (total absence) level in this study. Based on the analysis, the average total viable count in the three types of beverages tested exceeded the permissible level (*p* < 0.05), whereas 71% (a total of 22 samples) of samples also exceeded the permissible total coliform level.

For the *E. coli* parameter, only one sample was contaminated by this bacterium; it was a milk-based drink with an average of 3.05 log CFU/mL which exceeded the standard value that requires a total absence of *E. coli* in any 100 mL drinking water as stipulated by WHO Guidelines for Drinking-Water Quality [12]. Based on the FSANZ Compendium of Microbiological Criteria for Food [13], the average *S. aureus* counts for milk-based drinks and fruit juices are considered unsatisfactory whereas the average reading for cordial-based drinks was categorized as marginal (unsatisfactory: log 3 to ≤log 5; marginal: log 2 to <log 3 CFU/mL).

The one-way ANOVA tests conducted showed no significant difference (*p* < 0.05) between the contaminant levels of total viable count, total coliform, and *S. aureus* in the three types of beverages tested. A one-way ANOVA test was not performed for the *E. coli* parameter because only one sample was found to be a positive sample.

## 4. Discussion

Street food offers a highly convenient source of low-cost food for the public and serves as part of a tourist attraction in some countries [14]. However, this commodity also contributes to the risks of foodborne diseases if food safety and hygiene measures are not considered [3]. In this study, the presence of total viable counts, total coliforms, *E. coli*, and *S. aureus* was assessed in three different types of beverages sold by street vendors. Total viable counts, total coliforms, *E. coli*, and *S. aureus* are often used as indicators of microorganisms in determining the level of hygiene practices in a food handling setting [15]. Total viable counts can be used to measure the level of overall bacterial contamination of food or beverage samples tested, whereas total coliform is a suitable indicator to detect fecal contamination in food products [16,17]. The level of average bacterial contamination in the beverage samples tested in this study ranged from 4.90–5.30 log CFU/mL which was considerably higher compared to the level of bacterial contamination found on street-vended snacks and local cakes (kuih) around Chow Kit area which reportedly ranged from 3.88–4.07 mean log CFU/mL [10]. A study by Chong et al. [18] on fruit juices also showed a lowered average TVC in comparison with the levels of contamination found in the current study. However, high levels of microbial load in street-vended beverages were observed in studies conducted in other countries [19,20]. Milk-based drinks were identified to have the highest microbial load in this study. This finding is in accordance with a previous study conducted on milk tea drinks in Taiwan that observed total viable counts as high as >5.4 log CFU/mL [21]. High levels of microbial load may indicate a higher risk of the presence of pathogenic bacteria in the drink [19].

The presence of total coliform in street-vended beverages in this study indicates that the level of cleanliness and safety of beverages is not at par. According to a study conducted by Nemo et al. [22], the presence of coliforms in ready-to-eat foods or beverages sold by street vendors may be due to fecal contamination in food that occurs during and immediately after the preparation process. The presence of this bacteria could also be due to the use of unboiled water during the production process of beverages, water used to wash the vending utensils, or direct contamination of the utensils either from the hands or body of the food handlers themselves [7,22]. Coliform bacteria have the potential to divide into large amounts when contaminated food is left at ambient temperature for a while [23]. In addition, unhygienic environmental conditions of the vending site could also contribute to contamination by these microorganisms [19].

*E. coli* was also an indicator bacteria for fecal contamination in drinks to identify food and beverage samples that may contain unacceptable levels of fecal contamination [12]. *E. coli* was considered to be a more specific indicator bacteria to detect fecal contamination compared to fecal coliform because the most common test for fecal coliform also detects heat-resistant non-coliform bacteria [24]. The presence of *E. coli* can also indicate the presence of pathogenic *E. coli* in food products. Food contaminated by enterohemorrhagic *E. coli* (EHEC) O157:H7 may cause severe illnesses to humans such as bloody diarrhea or hemolytic uremic syndrome even at low doses (10–100 cells) [25]. In this study, although only one sample (milk-based drink) was found to be contaminated by *E. coli*, the level of contamination was very high (>log 3 CFU/mL).

The presence of flies at the beverage vending site was likely the cause of this bacterial contamination. According to Kateryn [26], flies are vectors that attempt to be the source of *E. coli* contamination. The same study found that *E. coli* can survive in the larvae of flies and gut of an adult fly. Based on observations of the surroundings of the street-vended beverages vending site around Chow Kit, almost every stall had garbage bins located near their booth. The trash cans are often uncovered and are sites for flies. In addition, a low level of personal hygiene practices such as failure of the food handlers to wash their hands properly could also be a contributing factor for *E. coli* contamination in sold products [27].

Previous studies have shown that fruit juices are often contaminated by pathogenic bacteria which have resulted in a foodborne epidemic [28]. Among the pathogenic bacteria that often contaminate fruit juice is enterohemorrhagic or Shiga-toxin-producing *E. coli* O157:H7 as well as various *Salmonella* serotypes. Typically, juices have low acidity between pH 4.8–6.2, which is particularly suitable for the growth of pathogenic bacteria [29]. *S. aureus* is one of the main bacterial agents that causes foodborne illness in humans [30]. The presence of *S. aureus* bacteria in this study clearly proved that the level of hygiene practiced by the street vendors was unsatisfactory. The cause of *S. aureus* contamination can either originate from the cleanliness of the vending site or the attitude of the beverage handlers [31]. Bacteria enterotoxin-producing *S. aureus* is often found in the nose, throat, and skin. *S. aureus* contamination can occur through direct contact with hands or respiratory secretion [32]. Therefore, food handlers can be considered as the main vectors of *S. aureus* contamination in food.

Based on our findings, the presence of *S. aureus* in fruit juices was slightly higher compared to other types of tested drinks. In the current study, almost all of the observed street food vendors around the Chow Kit area did not use gloves while handling beverages indicating that *S. aureus* contamination of the beverages most likely occurred from cross-contamination from the street vendors’ bare hands. The cross-contamination event from the handlers’ hands may be a result of the manual process of peeling fruit skins and juicing, thus confirming that the street food vendors did not wear gloves during this process. Fruits that are often used to produce juices do not involve heat-processing and do not contain preservatives. Therefore, these fruits are easily infected by bacteria and fungi [33]. Other sources of contamination of *S. aureus* in fruit juices include equipment used during beverage handling and fruit juice processing as reported by Chong et al. [18] who found the presence of *Staphylococcus* species on fruit juice extractors as well as utensils used to cut the fruit, which contributed to the contamination of the produced fruit juices.

Other sources of microbial contamination in street-vended beverages were the occurrence of cross-contamination between food or surfaces contaminated by bacteria with the beverages. Food contact surfaces may still harbor a high number of bacterial cells despite their clean appearances [34]. All street food vendors should ensure that all equipment, utensils, and other food contact surfaces that are regularly cleaned and kept in hygienic conditions during their operations [1]. Most food or beverage handlers handle food or beverages without using gloves. At the same time, they also handle money [35]. This can lead to pathogenic microorganism contamination of beverages caused by the unsafe food handling behavior of the beverage handlers. Lack of glove-wearing practices by the food handlers may be due to lack of food safety knowledge or their attitude towards embracing the food safety culture [27]. It is a mandatory requirement for every food handler in Malaysia to attend the food handling course organized by Ministry of Health agencies before they can be involved in any food handling procedures [36]. However, the level of food safety knowledge among the street food vendors involved in this study, their level of attitude towards safe food practices, and their status of attendance at the mandatory food handling course were not known in this study. Hence, the relation between their knowledge and the level of bacterial contamination found in their merchandise could not be distinctly determined.

Apart from that, bacterial contamination in street-vended beverages was also a result of using ice blocks. A significant increase in total viable count of contamination in beverages has been previously reported. This contamination occurs from time to time during the cooling of beverages by merely adding ice cubes [37]. The results of microbiological safety assessment made by Sunday et al. [38] found that pathogenic bacteria contaminated all samples of edible ice used by street vendors. Ice cubes can be contaminated during their production and transportation by the ice distributors [37]. According to the Malaysian Food Act [36], all food handlers must obtain ice cube supplies only from licensed manufacturers. Improper storage of ice cube at the vending sites by the street vendors could also contribute to the contamination of the ice cubes. In addition, there was an absence of temperature control at the site to maintain the ice, and often vendors’ booths will be exposed to high ambient temperatures that can promote the growth of microorganisms [3].

## 5. Conclusions

In conclusion, the microbiological safety of beverages sold by street vendors in Chow Kit was average. The level of bacterial contamination exceeded the standards set by the Malaysian specifications for ready-to-drink beverages (carbonated and non-carbonated) (MS601:1994), WHO Guidelines for Drinking-Water Quality, and they fall under the marginal and unsatisfactory category based on the FSANZ Compendium of Microbiological Criteria for Food. This study suggests that food safety education needs to be emphasized among food handlers and the provision of adequate sanitary facilities at vending sites is highly needed. Public awareness concerning how to differentiate between safe and unsafe street food vendors is also essential. This will help to maximize the quality of the beverages sold at the vending sites and thus increase consumers’ confidence regarding street food safety.

## Figures and Tables

**Table 1:** Total microbial load of total viable count (TVC), total coliform, *Escherichia coli*, and *Staphylococcus aureus* in street-vended beverages from Chow Kit.

Bacteria Loads in Beverages								
Types of Beverages	TVC		Total Coliform		*Escherichia coli*		*Staphylococcus aureus*	
	No. (%) ^1^	Average ± SD ^2^	No. (%)	Average ± SD	No. (%)	Average ± SD	No. (%)	Average ± SD
Cordial-based drinks (*n* = 11)	9 (81.8)	4.90 ± 1.42	5 (45.5)	4.45 ± 0.75	0	0	5 (45.5)	2.82 ± 0.46
Milk-based drinks (*n* = 9)	9 (100)	5.30 ± 1.11	9 (100)	4.30 ± 1.13	1(11.1)	3.05 ± 0	7 (77.8)	3.29 ± 0.86
Fruit juices (*n* = 11)	10 (90.9)	4.90 ± 0.87	8 (72.7)	4.75 ± 0.79	0	0	6 (54.5)	3.42 ± 1.15
Total (*n* = 31)	28 (90.3)	5.03 ± 0.23	22 (71)	4.50 ± 0.23	1 (3.2)	3.05 ± 0	18 (58.1)	3.18 ± 0.32

^1^ Number of positive samples. ^2^ Mean bacterial counts expressed in log Colony Forming Unit/mL (CFU/mL). SD: standard deviation.

## References

[1] Codex Alimentarius. CXC76R-2017 Regional Code of Hygienic Practice for Street Vended Foods in Asia. http://www.fao.org/fao-who-codexalimentarius/sh-proxy/en/?lnk=1&url=https%253A%252F%252Fworkspace.fao.org%252Fsites%252Fcodex%252FStandards%252FCXC%2B76R-2017%252FCXP_076Re.pdf.

[2] Tambekar D.H., Jaiswal V.J., Dhanorkar D.V., Gulhane P.B., Dudhane M.N. (2009). Microbial quality and safety of street vended fruit juices: A case study of Amravati City. J. Food Saf..

[3] Alimi B.A. (2016). Risk factors in street food practices in developing countries. Food Sci. Hum. Wellness.

[4] Azis A., Tek L.X. (2018). Community perceptions of street hawkers in Maaysia. Sumatra J. Disastergeogr. Geogr. Education..

[5] Barro N., Bello A., Aly S., Ouattara C., IIboudo A., Traaore A. (2006). Hygienic status assessment of dishwashing waters, utensils, hands and pieces of money from street food processing sites in Ouagadougou (Burkina Faso). Afr. J. Biotechnol..

[6] Das A., Nagananda G.S., Bhattacharya S., Bhardwaj S. (2010). Microbiology quality of street-vended indian chaats sold in Bangalore. J. Biol. Sci..

[7] Auad L.I., Ginani V.C., Stedefeldt E., Nakano E.Y., Nunes A.C.S., Zandonadi R.P. (2019). Food safety knowledge, attitudes and practices of Brazilian food truck food handlers. Nutrients.

[8] Odeyemi O.A., Sani N.A. (2016). Antibiotic resistance and burden of foodborne diseases in developing countries. Future Sci. OA.

[9] Tasnim F., Hossain M.A., Nusrath S., Hossain M.K., Lopa D., Haque K.M.F. (2010). Quality Assessment of Industrially Processed Fruit Juices Available in Dhaka City, Bangladesh. Malays. J. Nutr..

[10] Rahim N.L.A., Bakar N.F.A., Zulfakar S.S. (2019). Bacterial contamination in foods sold by street vendors in Chow Kit, Kuala Lumpur. APEOHJ.

[11] Department of Standards Malaysia (1994). Specification for Ready-To-Drink Beverages (Carbonated and Non-Carbonated) (MS 601:1994).

[12] WHO (2017). Guidelines for Drinking-Water Quality.

[13] Food Standards Australia New Zealand (2016). Compendium of Microbiological Criteria for Food.

[14] Privitera D., Nesci F.S. (2015). Globalization vs. local. The role of street food in the urban system. Procedia Econ. Financ..

[15] WM W.M. (2014). Evaluation of environmental hygiene and microbiological status of selected primary school canteens. Health Environ. J..

[16] Abu Bakar I., Abdul Rafa A.A., Sani N.A. (2018). Microbiological quality evaluation of fried rice sold at food premises in Kuantan city, Pahang. Int. J. Allied Health Sci..

[17] Khan M.M., Islam M.T., Chowdhury M.M.H., Alim S.R. (2015). Assessment of microbiological quality of some drinks sold in the streets of Dhaka University Campus in Bangladesh. Int. J. Food Contam..

[18] Chong S.Y., Rao P.V., Soon J.M. (2017). Identification of *Escherichia* spp. strains in street-vended beverages and associated preparation surfaces using 16S rRNA analysis. Int. Food Res. J..

[19] Afreen A., Ahmed Z., Ahmad H., Khalid N. (2019). Estimates and burden of foodborne pathogens in RTE beverages in relation to vending practices. Food Qual. Saf..

[20] Simforian E., Nonga H.E., Ndabikunze B.K. (2015). Assessment of microbiolofical quality of raw fruit juice vended in Dar es Salaam city, Tanzania. Food Control..

[21] Lin C., Yang C., Chen P., Liu K., Lin H., Lin C., Lee Y., Cheng W., Wei C., Tsai Y. (2019). Assessment of microbiological and chemical quality of bubble tea beverages vended in Taiwan. J. Food Prot..

[22] Nemo R., Bacha K., Ketema T. (2015). Microbiological quality and safety of some-street vended foods in Jimma Town, Southwestern Ethiopia. Afr. J. Microbiol. Res..

[23] Craven H.M., Eyles M.J., Davey J.A., Hocking A.D. (2003). Enteric iNdicator Organisms in Foods. Foodborne Microorganisms of Public Health Significance.

[24] Odonkor S.T., Ampofo J.K. (2013). *Escherichia coli* as an indicator of bacteriological quality of water: An Overview. Microbiol. Res..

[25] Shah M.K., Aziz S.A., Zakaria Z., Lin L.C., Goni M.D. (2018). A review on pathogenic *Escherichia coli* in Malaysia. Adv. Anim. Vet. Sci..

[26] Kateryn R. (2003). Persistence and Significance of *E. coli* in House Flies (*Musca domestica*) and Stable Flies (*Stomoxys calcitrans*). Master’s Thesis.

[27] Sani N.A., Siow O.N. (2014). Knowledge, attitudes and practices of food handlers on food safety in food service operations at the Universiti Kebangsaan Malaysia. Food Control..

[28] Parish M.E., Heredia N., Wesley I., Garcia S. (2008). Food sAfety Issues and the Microbiology of Fruit Beverages and Bottled Water. Microbiologically Safe Foods.

[29] Tribst A.A.L., Sant’Ana A.S., Massaguer P.R. (2009). Review: Microbiological quality and safety of fruit juices-past, present and future perspectives. Crit. Rev. Microbiol..

[30] Gutierrez D., Delgado S., Sanchez D.V., Martinez B., Cabo M.L., Rodriguez A., Herrera J.J., Garcia P. (2012). Incidence of *Staphylococcus aureus* and analysis of associated bacterial communities on food industry surfaces. Appl. Environ. Microbiol..

[31] Ibekwe A.C., Okonko I.O., Onunkwo A.U., Donbraye E., Babalola E.T., Onoja B.A. (2008). Baseline *Salmonella* agglutinin titres in apparently healthy freshmen in Awka, South Eastern, Nigeria. Sci. Res. Essays..

[32] Argudin M., Mendoza M.C., Rodicio M.R. (2010). Food Poisoning and *Staphylococcus aureus* Enterotoxins. Toxins.

[33] Estrada C.S.M.L., Alcaraz E., Satorres S.E., Manfredi E., Velazquez L.C. (2014). Presence of enterotoxigenic *Staphylococcus aureus* in artisan fruit salads in the city of San Luis, Argentina. Braz. J. Microbiol..

[34] Zulfakar S.S., Sahani M., Hamid N.H.A. (2018). Microbiological assessment of food contact surfaces in residential college cafeterias at a local university in Malaysia. J. Sains Kesihat. Malays..

[35] Michaels B. (2002). Handling money and serving ready-to-eat foods. Food Serv. Tech..

[36] International Law Book Services (1983). Malaysia Food Act (Act 281) & Regulations.

[37] Agbaje L., Julius K., Oloke E.B., Gueguim K., Esther P. (2006). The microbiological quality of ice used to cool drinks and foods in Ogbomoso Metropolis, Southwest. Nigeria. Int. J. Food Saf..

[38] Sunday P.U., Nyaudoh U.N., Etido J.U. (2011). Microbiological quality and safety evaluation of fresh juices and edible ice sold in Uyo Metropolis, South-South, Nigeria. Int. J. Food Saf..

